# How students perceive medical competences: a cross-cultural study between the Medical Course in Portugal and African Portuguese Speaking Countries

**DOI:** 10.1186/1472-6920-11-24

**Published:** 2011-05-25

**Authors:** Joselina Barbosa, Milton Severo, Mário Fresta, Mamudo Ismail, Maria Amélia Ferreira, Henrique Barros

**Affiliations:** 1 Center for Medical Education, Faculty of Medicine, University of Porto, (Al. Prof. Hernâni Monteiro), Porto, (4200-319), Portugal; 2 Department of Hygiene and Epidemiology, Faculty of Medicine, University of Porto, (Al. Prof. Hernâni Monteiro), Porto, (4200-319), Portugal; 3 Center for Advanced Studies in Medical Education, Faculty of Medicine, University Agostinho Neto, (Av. Hoji ya Henda), Luanda, (116), Angola; 4 Faculty of Medicine, University Eduardo Mondlane, (Av. Salvador Allende), Maputo, (257), Mozambique; 5 Institute of Anatomy, Faculty of Medicine, University of Porto, (Al. Prof. Hernâni Monteiro), Porto, (4200-319), Portugal; 6 Institute of Public Health, University of Porto, (Rua das Taipas), Porto (4050-600), Portugal

## Abstract

**Background:**

A global effort has been made in the last years to establish a set of core competences that define the essential professional competence of a physician. Regardless of the environment, culture or medical education conditions, a set of core competences is required for medical practice worldwide. Evaluation of educational program is always needed to assure the best training for medical students and ultimately best care for patients. The aim of this study was to determine in what extent medical students in Portugal and Portuguese speaking African countries, felt they have acquired the core competences to start their clinical practice. For this reason, it was created a measurement tool to evaluate self-perceived competences, in different domains, across Portuguese and Portuguese-speaking African medical schools.

**Methods:**

The information was collected through a questionnaire that defines the knowledge, attitudes and skills that future doctors should acquire. The Cronbach's Alpha and Principal Components Analysis (PCA) were used to evaluate the reliability of the questionnaire. In order to remove possible confounding effect, individual scores were standardized by country.

**Results:**

The order of the domain's scores was similar between countries. After standardization, Personal Attitudes and Professional Behavior showed median scores above the country global median and Knowledge alone showed median score below the country global median. In Portugal, Clinical Skills showed score below the global median. In Angola, Clinical Skills and General Skills showed a similar result. There were only significant differences between countries in Personal Attitudes (p < 0.001) and Professional Behavior (p = 0.043).

**Conclusions:**

The reliability of the instrument in Portuguese and Portuguese-speaking African medical schools was confirmed. Students have perceived their level of competence in personal attitudes in a high level and in opposite, knowledge and clinical skills with some weaknesses.

## Background

During the last years a global effort has been made to define the minimum essential requirements and core competences that all medical graduates worldwide must have to be good physicians [1-5]. These competences comprise not just a set of general knowledge but also a set of clinical skills and professional attitudes necessary to efficiently perform the medical practice [6]. Medicine is a global profession and medical competences, research and education have crossed national borders. A set of core competences required for medical practice throughout the world have been defined [1-5]. This concept is linked to the European Credit Transfer System (ECTS) and its impact on a global comparability in medical education. In Europe, the quality assurance frameworks and the portability of qualifications follow the directives of Bologna Process [7]. African Universities and its Medical Schools, supported by internal and external organizations, are trying to cope with this paradigm, changing their degree and qualification systems within European reforms [8-11]. Despite objective difficulties, such as lack of resources and properly delineated quality parameters to ensure quality university education [12], Medical Schools are being set up in many universities on the model of European reforms and offer the opportunity to change the current isolation and limited human resources [13].

A strict evaluation of the curriculum and educational reform are always needed to make sure that the best is done for medical students and ultimately for patients [14]. However, there is little or none information about how students of African medical schools evaluate their core competences. Against this background, the objectives of this study were to (i) create a measurement tool to evaluate self-perceived competences in different domains across Portuguese and Portuguese-speaking African medical schools and (ii) determine in what extent medical students in Portuguese and Portuguese-speaking African countries, felt they have acquired the core competences to start their clinical practice.

## Methods

In order to develop this research we have selected, in the African Portuguese-speaking countries: the Faculty of Medicine of the University Agostinho Neto (FMUAN), Angola and the Faculty of Medicine of the University Eduardo Mondlane (FMUEM), Mozambique with traditional curricula according to the current curricular reform set up with new directives. These are Institutions in a Global Program in Educational Development. In Angola, the medical school has a curriculum reform in progress since 2002 [10,11,15-17] and in Mozambique and Angola a joint project entitled "A NAME for Health - A Network Approach in Medical Education for the Pursuit of Quality of Higher Education Institutions and Health Systems" is under development since 2008 and it also focuses competences assessment.

All students attending the final year (6th year) of Medicine Course at FMUP - Portugal, FMUAN - Angola and FMUEM - Mozambique were invited to participate in a cross-sectional study. Among the 398 eligible students, 76 (77.6%) of Angola, 66 (67.3%) of Mozambique and 157 (77.8%) of Portugal, have participated in this study.

An evaluation scale of acquisition of core competences was designed based on "The Medical Graduate in Portugal" [18]. This document defined 112 competences combined in five domains (Knowledge, Professional Attitudes and Behavior, Clinical Skills and Practical Procedures, Communication Skills and General Skills).

All items followed a Likert scale ranging from 0 to 6, where: 0 - has not acquired any competence, 1 - has acquired the minimum competences... 6 - has acquired the maximum competences.

Principal Components Analysis (PCA) was used to evaluate the dimensionality of the questionnaire items and Direct Oblimin rotation was applied to simplify the factor loadings structure. Given the large number of items comprising the questionnaire, the PCA was performed by domain and not by the total items. The Scree Plot criterion [19] was used to determine the number of components. It was considered that items with absolute factor loadings of 0.35 or greater are interpreted as having a meaningful part on the whole domain.

The PCA was performed within country to observe if factor loadings structure was different by country. The internal consistency was assessed using Cronbach's Alpha [20].

The score of each domain was obtained by averaging the corresponding items divided by 6 and multiplied by 100 (the score ranged 0 to 100). The highest values scores represent the higher levels of competence.

The scoring and interpretation of the students' level of competence was based on medians.

To remove possible confounding effect by a particular country, all individual scores were standardized. The standardization was performed subtracting the country global median from each individual score and then dividing the difference by the country inter-quartile range. This calculation will enable to evaluate if competences follow the same pattern in all countries. That is, the domains with higher and lower scores were, respectively, higher and lower scores in all countries.

The Friedman test was used to compare the domain's scores in each country. Multiple comparisons were also carried out using the Wilcoxon test by adjusting the level of significance according to the Bonferroni correction for multiple comparisons.

The Kruskal Wallis test was used to compare the scores of each domain by countries. The Spearman correlation was used to evaluate the association between countries.

All statistics analyses were performed with the Predictive Analytics Software version 18.0 (PASW Statistics Software). The significance level was set at 0.05.

The ethical principles to this research followed the guidelines approved by FMUP, FMUAN and FMUEN.

## Results

### Participants Characteristics

In Angola, Mozambique and Portugal 15.8%, 60.6% and 91.7% of the students were aged less than or equal to 25 years old, respectively. Regarding the distribution by gender, in Angola, Mozambique and Portugal, 55.3%, 49.1% and 71.3% of the students were female, respectively (Table 1).

**Table 1:** Characteristics of students enrolled in the 6th year of the Courses of Medicine by country

	Country		
	**Angola**	**Mozambique**	**Portugal**

	**N (%)**	**N (%)**	**N (%)**

Age			
≤ 25	12 (15.8)	40 (60.6)	144 (91.7)
]25-30]	32 (42.1)	20 (30.3)	12 (7.6)
]30-61]	32 (42.1)	6 (9.1)	1 (0.6)
Gender			
Female	42 (55.3)	27 (49.1)	112 (71.3)
Male	34 (44.7)	28 (50.9)	45 (28.7)

### Reliability of the Questionnaire

#### Knowledge

The Scree Plot of the original scale "Knowledge" suggested the existence of one component to Angola explaining 44.4% of the variance; one component to Mozambique explaining 49.0% of the variance; and one component to Portugal explaining 48.7% of the variance. The PCA has indentified in all medical schools the same domain designated *Knowledge *(Cronbach's Alpha = 0.962) (Table 2).

**Table 2:** Principal Component Analysis

Domains	Angola			Mozambique			Portugal		
	Variance Explained	Minimum Factor Loadings	Maximum Factor Loadings	Variance Explained	Minimum Factor Loadings	Maximum Factor Loadings	Variance Explained	Minimum Factor Loadings	Maximum Factor Loadings
Knowledge	44.4	0.440	0.773	49.0	0.571	0.826	48.7	0.516	0.780
Personal Attitudes	40.0	0.462	0.860	47.8	0.452	0.849	59.2	0.589	0.855
Professional Behavior	14.5	0.388	0.901						
Clinical Skills	61.8	0.662	0.898	59.0	0.536	0.873	56.2	0.586	0.844
Communication Skills	66.3	0.539	0.897	59.5	0.562	0.871	66.7	0.654	0.897
General Skills	62.3	0.575	0.900	64.6	0.664	0.895	60.4	0.531	0.884

#### Professional Attitudes and Behavior

The Scree Plot of the original scale "Professional Attitudes and Behavior" suggested the existence of two components to Angola explaining 50.4% of the variance (the first explaining 40.0% and the second explaining 14.5%); one component to Mozambique explaining 47.8% of the variance; and one component to Portugal, explaining 59.2% of the variance.

A PCA followed by direct oblimin rotation was carried out in Angola. The sub-scales "Professional Relationships" and "Relation with the Society and with the System of Health Care" were joined in one component designated *Professional Behavior *(Cronbach's Alpha = 0.917). The sub-scale "Personal Attributes" was split in two components, so it was decided to considerate this domain *Personal Attributes *(Cronbach's Alpha = 0.915) with only the items that load in second component. Therefore, not being considered in the analysis the four items that load in first component ("Recognize my own limitations", Personal commitment in the defense of professional values", "Take responsibility for my learning", "Conduct a self-reflection").

Based on Angola PCA structure, the items were separated in two domains for all medical schools (Table 2).

#### Clinical Skills and Practical Procedures

The Scree Plot of the original scale "Clinical Skills and Practical Procedures" suggested the existence of one component to Angola explaining 61.8% of the variance; one component to Mozambique explaining 59.0% of the variance; and one component to Portugal explaining 56.2% of the variance. The PCA has indentified in all medical schools the same domain designated *Clinical Skills *(Cronbach's Alpha = 0.970) (Table 2).

#### Communication Skills

The Scree Plot of the original scale "Communication Skills" suggested the existence of one component to Angola explaining 66.3% of the variance; one component to Mozambique explaining 59.5% of the variance; and one component to Portugal explaining 66.7% of the variance. The PCA has indentified in all medical schools the same domain designated *Communication Skills *(Cronbach's Alpha = 0.970) (Table 2).

#### General Skills

The Scree Plot of the original scale "General Skills" suggested the existence of one component to Angola explaining 62.3% of the variance; one component to Mozambique explaining 64.6% of the variance; and one component to Portugal explaining 60.4% of the variance. The PCA has indentified in all medical schools the same domain designated *General Skills *(Cronbach's Alpha = 0.955) (Table 2).

### Core Competences

**Figure 1:**
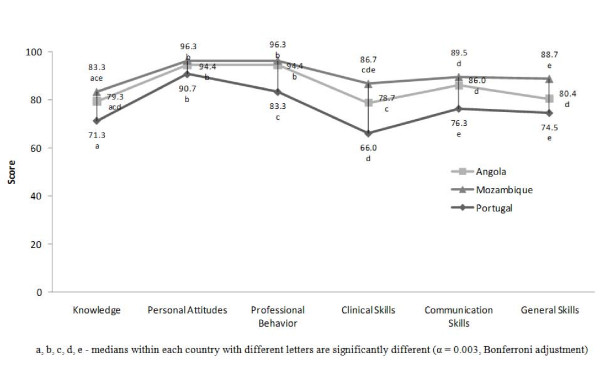
**Self-Perceived competences of students from Angola, Mozambique and Portugal (0 to 100 points).** Medians within each country with different letters are significantly different (α = 0.003. Bonferroni adjustment).

The scores in all domains and in all medical schools ranged from 66.0 to 96.3 points. The domains with higher scores in Angola and Mozambique were *Personal Attitudes *(94.4 and 96.3 points, respectively) and *Professional Behavior *(94.4 and 96.3 points, respectively). In Portugal it was *Personal Attitudes *(90.7 points). The domains with lower scores in Angola were *Clinical Skills *(78.7 points) and *Knowledge *(79.3 points). In Mozambique it was *Knowledge *(83.3 points), *Clinical Skills *(86.7 points) and *General Skills *(88.7). In Portugal it was *General Skills *(74.5 points) and *Communication Skills *(76.3 points). Mozambique medical students showed higher scores in all competences than medical students from Angola and Portugal; those from Angola presented a higher level of competences than their Portuguese colleagues (Figure 1).

There were significant correlations between the domain's scores assigned by each country (Portugal vs. Angola r = 0.986; Portugal vs. Mozambique r = 0.928; Angola vs. Mozambique r = 0.941).

To remove possible confounding effects by country, all individual scores were standardized. After standardization, significant differences between countries were only detected in the competences: *Personal Attitudes *(p < 0.001) and *Professional Behavior *(p = 0.043). Portugal showed significantly higher scores than Mozambique in *Personal Attitudes *(0.66 vs. 0.32), whereas Angola showed higher scores than Portugal and Mozambique (0.51 vs. 0.32).

In all countries, *Personal Attitudes *(Portugal = 0.66; Angola = 0.51; Mozambique = 0.32) and *Professional Behavior *(Portugal = 0.32; Angola = 0.51; Mozambique = 0.32) showed median scores above the country global median and *Knowledge *alone (Portugal = -0.24; Angola = -0.33; Mozambique = -0.42) showed median score below the country global median.

In Portugal, *Clinical Skills *(Median = -0.49) showed a score below the global median. In Angola, *Clinical Skills *(Median = -0.37) and *General Skills *(Median = -0.27) showed a similar result (Table 3).

**Table 3:** Distribution of domains by country (standardized values)

	Country						
	**Portugal**		**Angola**		**Mozambique**		

	**Median (p25-p75)**	*p *value*	**Median (p25-p75)**	*p *value*	**Median (p25-p75)**	*p *value*	***p *value^*+*^**

Knowledge	-0.24 (-0.64;0.19)	**< 0.001**	-0.33 (-0.85;0.05)	**0.001**	-0.42 (-0.85;-0.06)	**< 0.001**	0.075
Personal Attitudes	0.66 (0.23;0.92)	**< 0.001**	0.51 (0.20; 0.71)	**< 0.001**	0.32 (0.00; 0.53)	**< 0.001**	**< 0.001**
Professional Behavior	0.32 (-0.11; 0.75)	**< 0.001**	0.51 (-0.01; 0.71)	**< 0.001**	0.32 (-0.21; 0.53)	**0.001**	**0.043**
Clinical Skills	-0.49 (-0.98; -0.02)	**< 0.001**	-0.37 (-1.22; 0.11)	**0.002**	-0.23 (-0.96; 0.31)	0.061	0.189
Communication Skills	-0.01 (-0.50;0.040)	1.000	0.04 (-0.45;0.052)	0.903	-0.07 (-0.62;0.43)	0.156	0.445
General Skills	-0.09 (-0.57;0.32)	0.224	-0.27 (-0.77-0.11)	**0.001**	-0.11 (-0.90; 0.20)	0.245	0.104

* Median significantly different from zero

^+ ^Difference of medians between countries

## Discussion

At the end of graduation, medical students must possess the professional competences that are necessary to meet their individual and collective responsibilities to society. Acquired competences are definitely a central indicator for the quality of a curriculum. Regardless of the environment, culture or medical education conditions, a set of core competences are required for medical practice worldwide [1-5].

### How domains are perceived by medical students

In this sample, four original domains showed good homogeneity (strong first factor) and high internal consistency (minimum Alpha = 0.92) and only one domain (Professional Attitudes and Behavior) showed the existence of two sub-domains. Furthermore, these items and competences showed content validity based a previous study [18]. So, our scale evaluates competences combined in six domains: Knowledge, Personal Attitudes, Professional Behavior, Clinical Skills, Communication Skills and General Skills.

Overall, in all studied countries, medical students showed that they felt truly competent in the core set of medical competences. All students tended to give high scores to all domains.

Students from Mozambique showed higher scores than their colleagues from Angola and these showed higher scores than Portuguese medical students. This may show that the self-perceived competences are confounded by contextual differences. This outcome could be explained by different self confidence levels attributed to the different learning environments or countries [21]. Other factors can also contribute towards the acquisition of competences and its perception on students. These found factors and differences can be explained by possible differences in teaching and background of students among countries which will affect perceived competences. Correlation competences between countries showed similar patterns scores; the domains with higher and lower scores had, respectively, higher and lower scores in all countries. So, it was decided to standardize the individual scores by country to remove possible confounding effects. After standardization it was possible to understand the real differences. The domains *Personal Attitudes *and *Professional Behavior *were found to have higher scores of self-perceived competences in all countries. This means that medical students felt more competent in their attitudes. The domain *Communication Skills *was evaluated in the median. A study about perceptions of educational development during the first year of post graduation for medical students in the UK showed that when they were asked which competences they experienced a bigger development the students emphasized the skills of communication, teamwork and outcomes related to personal development [22]. These results may be due to the fact that students take these competences as inherent to a future physician and therefore assume that they must be acquired. It is noticed that there are also areas where the assessment is difficult. Methods and techniques of assessment in medical schools are based, is most cases, on written work and a discussion rather than a direct observation of student-patient interactions [23]. This could influence students in their perception based on their grades.

Instead, *Knowledge *in all countries and *Clinical Skills *in Portugal and Angola showed lower scores. Many studies have addressed their experiences as new doctors when they enter the world of clinical practice. It has been demonstrated that new graduate doctors often mention problems associated with their own shortcomings in knowledge, skills and general competences [24].

The low values assigned to the acquisition of competences in *Clinical Skills *may be due with the students' fear of making mistakes or the limited opportunities for practice during under graduation. This aspect emphasizes the importance of clinical training throughout the medical education process. Still, the area of therapeutics, included in *Clinical Skills*, causes a great discomfort to students of Medicine. At the end of the degree and prior to take on the responsibility for prescribing, students do not feel prepared and they are aware that prescribing has the power to bring great benefits but also the risk of causing major damage [25]. Most students will require not only knowledge of existing drugs but also a strong knowledge of the principles of pharmacotherapy sustained by a scientific knowledge of drug action.

Although *Personal Attitudes *and *Professional Behavior *were been the domains with higher scores in all countries, these were the only domains in which significant differences among countries were demonstrated. Portugal achieved a higher level of competences in *Personal Attitudes *which was significantly different from Mozambique. In Angola there was a higher level of competences in *Professional Behavior*, significantly different from Portugal and Mozambique.

### Limitations of the study

Albeit the relevance of the results obtained, this work shows limitations which are due to different factors that have to be considered for this discussion: (i) It must be pointed out that we obtained the samples students of different countries at different times, within educational contexts where differences in teaching patterns and backgrounds of students are likely to affect perceived competences. However, we tried to remove these confounding differences in perceived competences through the standardization. (ii) The method of self-assessment is neither objective nor free from beliefs and values that individuals hold about themselves. Self-evaluation instruments are more adequate to analyze work practices and promote reflection on performance [26]. However, they do not help at the same extent to judge the accuracy of the individual's evaluation. (iii) Despite knowing what a student think and what he is capable to do, we cannot provide data for the real performance.

## Conclusions

Self-assessment allows medical students not only to build self-reflection skills and to identify areas of improvement, but it also helps in decision-making and, therefore, it is an important part of medical education, towards patient care. Self-assessment is also a valuable tool to identify potential problem areas where either the objectives or teaching require adjustments [27].

This study provides some useful insights into the preparedness of students completing undergraduate medical education. Students had considered themselves with high level in all core competences, although with different levels of self-perceived competences. While the scores for the domains were different, the order of the scores was similar between countries. This result confirms the comparability among countries. However, to fully evaluate the quality of acquisition of medical competences, in terms to proceed towards accreditation process and consequently to provide solid background for comparability between medical graduates of different countries, it is necessary to take into consideration contextual and developmental differences.

The students have perceived their level of competence in personal attitudes in a high level, and in opposite, knowledge and clinical skills with some weaknesses. There is a need to evaluate attitudes and the conduct of medical students with direct measures.

The dynamics of changes in medical education is determined by multiple factors that need to be discussed and articulated as a whole. The evaluation of medical competence is a complex task, and no single evaluation tool can effectively assess the physician's knowledge, skills and attitudes [28]. Ongoing evaluations in student competences should be undertaken as well new strategies for learning should be implemented in anticipation of changes in environment and to create opportunities for acquisition of medical competences. There is a need for further research to explore how medical competence is best assessed; be it in written formats, such as case studies, self-evaluation or another format, such as observing student's performance.

Two aspects have to be followed in further researches: (i) a follow-up of postgraduates is important to obtain their perceptions on professional performance, as the change in the clinical context allows a broader view and more accurate evaluation of medical competences and (ii) the present study could offer baseline data for the global comparability in medical education in Portuguese-speaking countries and then provide the generalisability of the study.

## Competing interests

The authors declare that they have no competing interests.

## Authors' contributions

JB designed the study, carried out the design research, performed the statistical analysis and drafted the manuscript. MS participated in study design, helped in statistical analysis and helped to draft the manuscript. MF contributed with technical support and helped to draft the manuscript. MI contributed with technical support and helped to draft the manuscript. MAF conceived of the study, coordinated the research and made a critical review of the manuscript. HB made a critical review of the manuscript and helped to draft the final approval of the version to be published. All authors read and approved the final manuscript.

## Authors' Information

JB is researcher at the Faculty of Medicine of the University of Porto, graduate in Mathematics Applied to Technology. She attends the Master Course in Public Health. MS is researcher at the Faculty of Medicine of the University of Porto and Public Health Institute of the University of Porto, graduate in Mathematics Applied to Computation. He attends the PhD Course of Public Health. MF is Professor of Physiology and Director of the Center for Advanced Studies in Medical Education at the Faculty of Medicine of the University Agostinho Neto, Luanda, Angola. MI is Professor of Pathology and Director of the Faculty of Medicine of the University Eduardo Mondlane, Maputo, Mozambique. MAF is Full Professor and Director of the Center for Medical Education at the Faculty of Medicine of the University of Porto. HB is Professor of Hygiene and Epidemiology and Director of the Department of Hygiene and Epidemiology at the Faculty of Medicine of the University of Porto. He's also Director of the Institute of Public Health of the University of Porto and Coordinator of the National Commission for HIV AIDS.

## Pre-publication history

The pre-publication history for this paper can be accessed here:

http://www.biomedcentral.com/1472-6920/11/24/prepub
